# Meet the Editorial Board Member

**DOI:** 10.2174/1570159X2212240517230620

**Published:** 2024-05-17

**Authors:** Michel Baudry

**Affiliations:** 1 University Professor, Western University of Health Sciences, 309 E. 2^nd^ St Pomona, CA 91766, USA

   Dr. Baudry is currently a professor of Biomedical Sciences in the Graduate College of Biomedical Sciences at Western University of Health Sciences in Pomona, CA. He was also a professor of Biological Sciences, Neurology and Biomedical Engineering at USC, Los Angeles, CA. After graduating from the prestigious Ecole Polytechnique in Paris (France) in 1971, Michel Baudry obtained a Ph.D. in Biochemistry from the University of Paris VII in 1977. In 1978, he moved to the United States of America for a postdoctoral period with Prof. Gary Lynch at UC Irvine. He remained an assistant, then an associate professor at UCI, before moving to USC in 1989, where he worked until the end of 2011. Since 1989, Dr. Baudry is an associate member of the Center for the Neurobiology of Learning and Memory at UC Irvine, CA. Dr. Baudry is a world-known neuroscientist and has published over 400 manuscripts in peer-reviewed journals. The biochemical theory for Learning and Memory presented by him and Dr. Lynch in the early 80s is one of the most widely accepted theories in this field. Dr. Baudry’s research is directed at understanding the molecular mechanisms of learning and memory and those involved in neurodegenerative processes underlying numerous human brain diseases. Over the years, Dr. Baudry has made ground-breaking discoveries that have had a significant impact on neurosciences. In particular, he has demonstrated that learning and memory, and neurodegeneration share a number of mechanisms. Furthermore, he indicated that two variants of a calcium-dependent protease, calpain-1 and calpain-2, play opposite functions in the brain. Calpain-1 activation is required for learning, while calpain-2 limits the extent of learning. Calpain-1 is neuroprotective, but calpain-2 is neurodegenerative. In 2020, he was elected a fellow in the American Association for the Advancement of Sciences for his distinguished contributions to the field of molecular and translational neuroscience, in particular, to the understanding of the roles of calpain-1 and calpain-2 in synaptic plasticity and neurodegeneration. His laboratory has been continuously funded by various agencies since 1980.

   Dr. Baudry has also been involved in several biopharmaceutical start-up companies. In 1986, he co-founded Synaptics, Inc. In 1991, he co-founded Eukarion, Inc, which is now MindSet, Rx. In 2007, he co-founded Rhenovia Pharma, a drug discovery & development company located in Mulhouse, France. Finally, in February 2016, in collaboration with several faculty members at WesternU, he started NeurAegis, a neuroscience company directed at developing neuroprotective drugs for the treatment of a variety of neurodegenerative diseases. In particular, his current work is funded by a large grant from the Department of Defense to optimize a selective calpain-2 inhibitor for the treatment of traumatic brain injury. His current plan is to initiate clinical trials early in 2021 to test the safety and efficacy of the lead candidate calpain-2 inhibitor, NA-184.

   Dr. Baudry has been involved in numerous review panels of the NSF, the NIH, a number of international funding agencies and is on the editorial boards of several journals, including Neurobiology of Learning and Memory, Neural Plasticity (Chief-Editor), PlosOne, and Current Neuropharmacology.

## References

[1] Lynch G., Baudry M. (1984). The biochemistry of memory: A new and specific hypothesis.. Science.

[2] Morris R.G.M., Anderson E., Lynch G.S., Baudry M. (1986). Selective impairment of learning and blockade of long-term potentiation by N-methyl-aspartate receptor antagonist, AP-5.. Nature.

[3] Bi R., Broutman G., Foy M., Thompson R.F., Baudry M. (2000). The tyrosine kinase and MAP kinase pathways mediate multiple effects of estrogen in hippocampus.. Proc. Nat. Acad. Sci..

[4] Xu W., Wong T.P., Chery N., Gaertner T., Wang Y.T., Baudry M. (2007). Calpain-mediated truncation of mGluR1alpha: a key step in excitotoxicity.. Neuron.

[5] Zadran S., Qin Q., Bi X., Zadran H., Kim Y., Foy M.R., Thompson R.F., Baudry M. (2009). 17-ß-estradiol increases neuronal excitability through MAP kinase-induced calpain activation.. Proc. Nat. Acad. Sci. USA.

[6] Wang Y., Briz V., Chishti A., Bi X., Baudry M. (2013). Distinct roles for µ- and m-calpain in synaptic NMDAR-mediated neuroprotection and extrasynaptic NMDAR-mediated neurodegeneration.. J. Neurosci..

[7] Wang Y., Zhu G., Briz V., Hsu Y.-T., Bi X., Baudry M. (2014). A molecular brake controls the magnitude of long-term potentiation.. Nat. Commun..

[8] Baudry M., Bi X. (2016). Calpain-1 and calpain-2: the yin and yang of synaptic plasticity and neurodegeneration.. Trends Neurosci..

[9] Wang Y., Hersheson J., Lopez D., Ben, H M., Liu Y., Lee K.-H., Pinto V., Seinfeld J., Wiethoff S., Sun J., Amouri R., Hentati F., Baudry N., Tran J., Singleton A.B., Coutelier M., Brice A., Stevanin G., Durr A., Bi X., Houlden H., Baudry M. (2016). Defects in the *CAPN1* gene result in alterations in cerebellar development and in cerebellar ataxia in mice and humans.. Cell Reports.

[10] Wang Y., Liu Y., Lopez D., Lee M., Dayal S., Hirtado A., Bi X., Baudry M. (2017). Protection against TBI-induced neuronal death with post-treatment with a selective calpain-2 inhibitor in mice.. J. Neurotrauma.

